# Surface-Tension-Induced Phase Transitions in Freestanding Ferroelectric Thin Films

**DOI:** 10.1021/acs.nanolett.5c03216

**Published:** 2025-08-14

**Authors:** Svitlana Kondovych, Léo Boron, Franco N. Di Rino, Marcelo Sepliarsky, Anna G. Razumnaya, Anaïs Sené, Igor A. Lukyanchuk

**Affiliations:** † Institute for Theoretical Solid State Physics, 28394 Leibniz Institute for Solid State and Materials Research Dresden, Helmholzstr. 20, Dresden D-01069, Germany; ‡ Laboratoire de Physique de la Matière Condensée, 26993 Université de Picardie Jules Verne, 33 rue Saint Leu, Amiens 80039, France; ¶ Institute of Physics of the Czech Academy of Sciences, Na Slovance 2, Prague 8 182 00, Czechia; § Instituto de Fisica Rosario, CONICET-Universidad Nacional de Rosario, Ocampo y Esmeralda, S2000EKF, Rosario 2000, Argentina; ∥ 61790 Jozef Stefan Institute, Jamova Cesta 39, Ljubljana 1000, Slovenia

**Keywords:** Freestanding films, Ferroelectrics, Surface tension, Phase transition, Phase diagram

## Abstract

The control of ferroelectric topological phases in ultrathin films is central to the development of next-generation nanoelectronic devices. While epitaxial strain is widely used to tune polarization states, its applicability is inherently limited to substrate-bound systems. Here, we show that surface tension becomes a key mechanical factor in freestanding ferroelectric films, governing phase stability and the emergence of topological polarization textures. Using a thermodynamic free energy framework, we show that surface tension induces effective compressive stress in freestanding films, strongly influencing both uniform and topological polarization states. Surface tension also drives large-scale morphological instabilities, reshaping phase behavior in the ferroelectric regime. Our results reveal surface tension as a robust, substrate-independent mechanism for engineering polarization states in freestanding films, creating new opportunities for flexible, strain-free ferroelectric devices.

## Graphical abstract

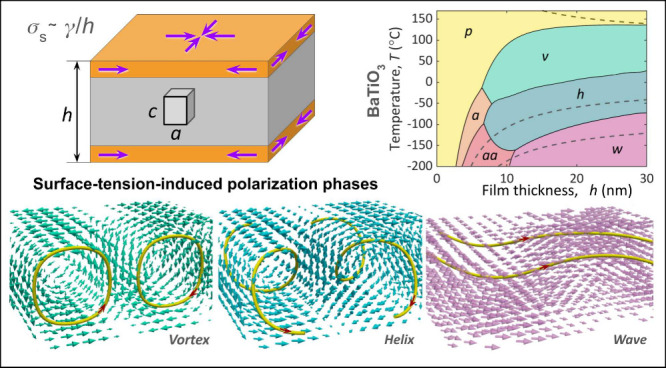

Ferroelectric thin films are essential for nanoscale electronic applications.^1,2^ Over the past decades, research has focused primarily on epitaxial thin films and superlattices grown on substrates using techniques such as pulsed laser deposition and molecular beam epitaxy. These systems benefit from epitaxial strain, providing a versatile platform to modify ferroelectric properties and explore strain-driven phase transitions, first predicted in refs 3,4 This approach advanced fundamental understanding and device performance, enabling further exploration of nanoscale ferroelectric behavior,^5,6^ including the formation of various topological structures.^7–12^ Recent progress in fabrication techniques, including sacrificial layer etching, water-soluble substrates, and van der Waals transfer, has enabled the manufacturing of high-quality freestanding ferroelectric thin films.^13^ This allows exploring the intrinsic properties of ferroelectrics not affected by substrate effects. However, studying freestanding films requires adapting methodologies developed for epitaxial systems, since detachment removes misfit strain as a thermodynamic driving parameter. Electrostatic and mechanical forces at free surfaces govern ferroelectric phase stability, domain morphology, and switching kinetics,^13–17^ enabling functionality control via surface design. In addition, experimental studies report a pronounced thickness dependence of polarization, dielectric response, and transition temperatures distinct from that observed in strained films,^18,19^ along with emergent topological states,^20–22^ presenting challenges to theoretical understanding.

In this Letter, we demonstrate that surface tension acts as a key thermodynamic factor in freestanding ferroelectric films. While commonly associated with liquids, surface tension also naturally arises at free surfaces of solids,^23,24^ including ferroelectrics,^25–27^ where it originates from broken intermolecular bonds and asymmetry of atomic environments. In freestanding films, this surface-induced stress becomes a driving parameter for polarization phase transitions, analogous to the substrate-induced strain in epitaxial films.

Studies on nanoscale ferroelectrics, nanorods and nanoparticles,^28–32^ revealed that surface tension can induce internal stress and govern the emergence of nontrivial polarization textures. Extending this concept to thin films, we consider surfaces of a freestanding film as elastic skin layers that generate additional stress, which causes intrinsic deformations and shapes ferroelectric and morphological behavior of the system. We estimate the magnitude of surface-tension-induced stress and study its impact on phase transitions and polar topological states in freestanding ferroelectric films. Surface-related effects were identified in earlier studies on relatively thick films ∼200 nm, where they were attributed to lattice mismatch with near-surface layers ∼20 nm.^33^ Building on this foundation, this work explores much thinner nanometric films, where surface tension arises as an intrinsic property of atomic-scale physical surfaces.

To quantify the role of surface tension in freestanding ferroelectric films, we estimate the surface tension constant γ, which defines the surface energy per unit area and the energetic cost of creating a free surface in a solid. We consider thin freestanding films of barium titanate BaTiO_3_ (BTO) and lead titanate PbTiO_3_ (PTO) as model ferroelectric systems. In PTO, γ_PTO_ ≈ 5.39 – 5.51 N/m was reported in ref 32. As for BTO, reported values of γ_BTO_ span a wide range 1–50 N/m, depending on the employed methodology and the specific system geometry.^25–28^ To refine this value, we perform atomic-level simulations of surface-induced lattice deformations in BTO, enabling a straightforward estimation of γ_BTO_. Importantly, we run the simulations in the paraelectric phase above the Curie temperature, isolating surface-induced deformations from those arising due to electrostriction in the ferroelectric phase.

We consider a freestanding film of thickness *h*, shown in Figure 1a. The free surfaces (orange) generate surface stresses σ (purple arrows), which induce deformation across the entire film volume (gray area). The total elastic energy of the film is expressed in terms of strain tensor components *u*_*ij*_ as the sum of surface, *E*_surf_, and volume, *E*_vol_, contributions,

E=Esurf+Evol=2γ(S0+ΔS)+12∫V(C11uii2+C12uiiujj)i≠jdV 1

**Figure 1:**
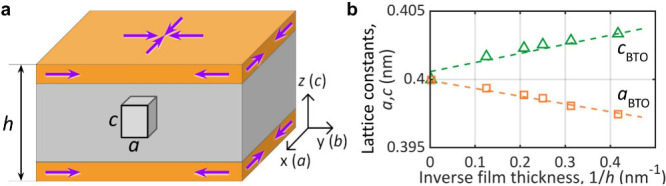
Surface tension in freestanding films. (a) Section of an infinite freestanding film of thickness *h*, with free surfaces (orange). Surface tension (purple arrows) induces compressive stress within the film. The initially cubic unit cell is shown in a deformed state, elongated along the vertical axis, with modified in-plane and out-of-plane lattice parameters *a* and *c*, respectively. The crystal axes (*a*, *b*, *c*) coincide with the coordinate axes (*x*, *y*, *z*), as shown on the right. (b) Estimation of the surface tension constant γ from the inverse thickness dependence of the in-plane (*a*) and out-of-plane (*c*) lattice parameters in freestanding BaTiO_3_ (BTO) films in the paraelectric phase. Markers show atomic-level simulation data; dashed lines represent corresponding linear fits.

Here and further, summation over repeated indices {*i*, *j*} = {*x*, *y*, *z*} (or {1, 2, 3}) is assumed, *S*_0_ is the surface area of a nondeformed film, $\Delta S=\int_{S}\left(u_{xx}^{s}+u_{yy}^{s}\right)ds$ is the surface-tension-induced shrinking of surface area with respect to *S*_0_, where strains $u_{ij}^{s}$ are taken at the surface. *C*_11_ and *C*_12_ are components of the stiffness tensor *C*_*ijkl*_ in Voigt notation, assuming the cubic symmetry of the material. The crystallographic *a*-, *b*-directions are oriented in the *x*–*y* plane of the film, and *c*-direction is along the *z*-axis.

Minimization of the energy (1) with respect to *u*_*ij*_ provides the resulting strains in the film,

2 uzz=4γhC12(C11−C12)(C11+2C12)uxx=uyy=−C112C12uzz

These strains correspond to elastic stresses σ_*xx*_ = *∂E*/*∂u*_*xx*_ = – 2γ/*h*, σ_*yy*_ = σ_*xx*_, and σ_*zz*_ = *∂E*/*∂u*_*zz*_ = 0. We further denote the surface-tension-induced stress σ_*s*_ ≡ σ_*xx*_ = σ_*yy*_,

3 $$\sigma_{s}=-2\gamma /h$$

and consider σ_*s*_ as the driving parameter of the system.

The elastic stability conditions of the crystal require *C*_11_ > *C*_12_ > – *C*_11_/2 and *C*_11_ > 0,^34^ which ensures that *u*_*xx*_ and *u*_*yy*_ are negative, resulting in lateral contraction of the film. In addition, *C*_12_ > 0 for BTO and PTO, leading to transverse expansion of the films.

Equations 2 enable estimation of γ by comparing the lattice parameters *a* and *c* in surface-tension-deformed freestanding films of different thicknesses with the corresponding bulk values, *a*_0_ and *c*_0_. The thickness-dependent lattice parameters in BTO films were obtained using atomistic simulations, following the approach described in ref^32^ (Methods section) with atomic potentials from ref^35^ For consistency, the same simulation set was used to extract the stiffness constants for BTO: *C*_11_ = 276.6 GPa, *C*_12_ = 123.9 GPa, and *C*_44_ = 143.4 GPa. The obtained values of *a* and *c* as functions of the inverse film thickness 1/*h* are shown in Figure 1b. As expected, lateral contraction of the film is accompanied by transverse expansion, both scaling as 1/*h*. By taking *u*_*xx*_ = *u*_*yy*_ = (*a* – *a*_0_)/*a*_0_ and *u*_*zz*_ = (*c* – *c*_0_)/*c*_0_ and fitting the data using (2), we obtain the surface tension constant, γ_BTO_ ≈  2.06–2.58 N/m.

Having determined γ for typical perovskite ferroelectrics, we explore how surface tension alters polar phases in freestanding films compared to substrate-strained systems. Since polarization states are highly sensitive to depolarization fields induced by bound charges, we consider two distinct types of electrostatic boundary conditions, short-circuit and open-circuit.

In the short-circuit case, film surfaces are assumed to be conducting and electrically connected, allowing free charges to fully compensate the polarization-bound charges. Such conditions may occur in films with ideal ultrathin electrodes, intrinsic surface conductivity (e.g., at oxide interfaces^36^), or under pressure-tuned oxygen chemical potential.^37^ As a result, the depolarization field is effectively suppressed and electrostatic contributions can be neglected. This leads to a uniform polarization distribution, unaffected by long-range electrostatics. Notably, misfit-strain-driven *a*_1_/*a*_2_ domains, typical for epitaxial films,^38–40^ are not generally expected to form in freestanding films.

In the absence of depolarization fields, uniform polar states are determined by minimizing the Ginzburg–Landau-Devonshire (GLD) functional, $F=\int_{V}FdV$ ,^3,41^ in which the polarization, *P*_*i*_ (*i* = {1, 2, 3}), and elastic stresses, σ_*ij*_, are the variational parameters, and the free energy density in notations of ref 42 is

4 $$F={\left[a_{i}P_{i}^{2}+a_{ij}P_{i}^{2}P_{j}^{2}+a_{ijk}P_{i}^{2}P_{j}^{2}P_{k}^{2}\right]}_{i\leq j\leq k}-Q_{ijkl}\sigma_{ij}P_{k}P_{l}-\frac{1}{2}s_{ijkl}\sigma_{ij}\sigma_{kl}$$

Here, *a*_*i*_ = *a*_*i*_(*T*), *a*_*ij*_ and *a*_*ijk*_ are the coefficient sets for the GLD functional,^43^ *Q*_*ijkl*_ is the stress-polarization coupling tensor, and $s_{ijkl}=C_{ijkl}^{-1}$ is the compliance tensor.

Considering the case of a freestanding film tuned by the uniform lateral stress σ_*s*_ (eq 3) and assuming a pseudocubic symmetry typical for BTO and PTO, we rewrite eq 4 as

5 F=a1*(Px2+Py2)+a3*Pz2−(s11+s12)σs2+[aijPi2Pj2+aijkPi2Pj2Pk2]i≤j≤ka1*=a1(T)−(Q11+Q12)σsa3*=a1(T)−2Q12σs

where the tensor components are given in Voigt notation, *s*_11_ = *s*_1111_, *s*_12_ = *s*_1122_, *Q*_11_ = *Q*_1111_, and *Q*_12_ = *Q*_1122_.

Minimization of eq 5 with respect to *P*_*i*_ allows determining uniform phases stabilized in freestanding films under different applied stresses σ_*s*_ and temperatures *T*. The corresponding σ_*s*_–*T* phase diagrams are analogous to the strain–temperature diagrams *u*_*m*_–*T*, developed for epitaxial films with misfit strain *u*_*m*_, known as Pertsev–Tagantsev (P–T) phase diagrams.^3,4^ Figure 2 compares the P–T phase diagrams *u*_*m*_–*T* for BTO (Figure 2a) and PTO (Figure 2b) with σ_*s*_–*T* phase diagrams obtained in the present study (Figure 2c and d). The transitions observed in σ_*s*_–*T* diagrams generally exhibit slight discontinuities, suggesting a first-order character.

**Figure 2:**
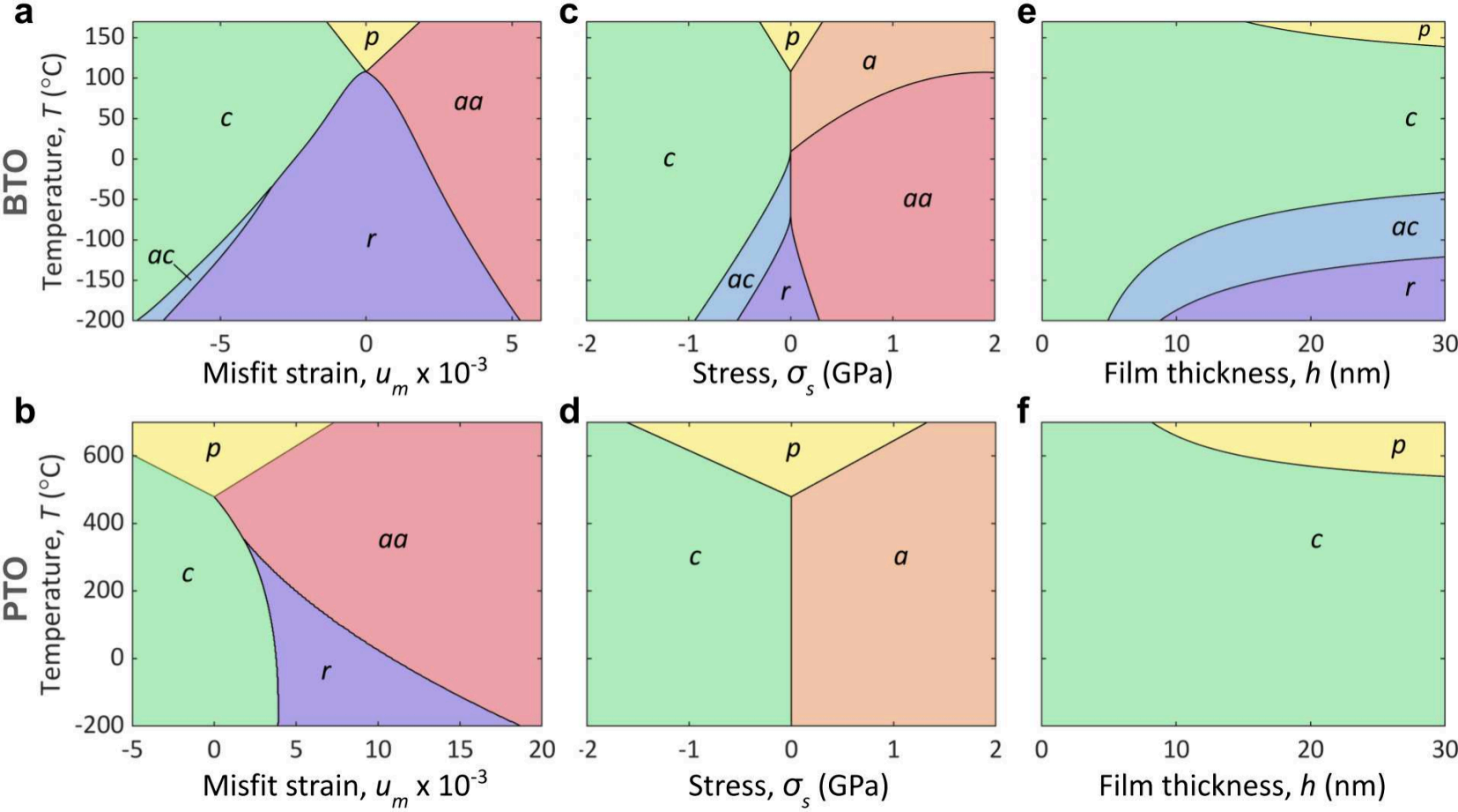
Uniform polarization phases in short-circuit BTO and PTO thin films. The structure of emerging *c*, *ac*, *r*, *a*, *aa*, and *p* phases is explained in the text. (a, b) Strain–temperature phase diagrams of epitaxial thin films of BTO (a) and PTO (b).^3,4^ (c, d) Stress–temperature phase diagrams of freestanding BTO (c) and PTO (d) films. (e, f) Thickness–temperature phase diagrams of freestanding BTO (e) and PTO (f) films driven by surface tension.

Interestingly, in addition to *c*-phase (0, 0, *P*_3_), *ac*-phase (*P*_1_, 0, *P*_3_), *r*-phase (*P*_1_, *P*_1_, *P*_3_), and *aa*-phase (*P*_1_, *P*_1_, 0), observed in strained BTO films, one more polar state, *a*-phase (*P*_1_, 0, 0), emerges in the respective σ_*s*_–*T* phase diagram. (*p*-phase corresponds to the paraelectric state (0,0,0) in all diagrams). In contrast, σ_*s*_–*T* diagram for PTO is reduced to just two states, *a*-phase and *c*-phase, while *aa*- and *r*-phases observed in *u*_*m*_-*T* diagram do not emerge. Notably, σ_*s*_–*T* diagrams in Figure 2c,d exhibit continuity with bulk phase transitions: at σ_*s*_ = 0 the cooling path mimics the bulk sequence cubic → tetragonal → orthorhombic → rhombohedral in BTO and cubic → tetragonal in PTO. A small tensile or compressive stress reshapes these sequences into strain-aligned variants, favoring in-plane *a*- and *aa*- or out-of-plane *c*- and *ac*-phases, respectively.

Establishing σ_*s*_–*T* phase diagrams enables exploration of polar phases in freestanding films, where surface tension induces stress via (3). This stress is always compressive (negative) across all thicknesses. Taking into account the inverse dependence of σ_*s*_ on *h* and using the values for γ specified above, we present *h*–*T* phase diagrams for BTO (Figure 2e) and PTO (Figure 2f).

In BTO films thicker than 10 nm three distinct ferroelectric phases consequently emerge upon cooling from the paralelectric state: *c*-phase, *ac*-phase, and *r*-phase. In thinner films, only *c*-phase is observed. However, in such ultrathin films, atomistic surface-induced decoherence can suppress the ferroelectric state;^44^ this lies beyond the scope of the current model. The *h*-*T* diagram for freestanding PTO films, Figure 2f, is more trivial and solely consists of one uniform polarization *c*-phase.

In ferroelectric films with open-circuit surfaces, the depolarization field induced by surface bound charges reshapes the polarization texture, leading to the domain formation to minimize the depolarization energy.^45–48^ The domains arrange into alternating vortex stripes or polarization bubbles, both structures substantially reducing the depolarization energy. The emergence of topological phases considerably modifies phase diagrams due to the strong inhomogeneity in **P**(*r*). Accurate determination of these phase diagrams requires advanced modeling approaches, such as atomistic or phase-field simulations,^49–55^ which are computationally demanding. Here, we employ a simplified technique, the soft-domain approach,^56^ which, though approximate, captures essential features of nonuniform phases, providing a sketch of the underlying phase diagram.

In this approach, the polarization distribution is represented by the first harmonic of its Fourier expansion, expressed through sine and cosine functions. The applicability of this method was numerically justified in ref 56, and experimental observations of the polarization profile^57^ indicate that it indeed closely resembles harmonic functions. In general form, the polarization soft-domain distribution in competing phases emerging in thin films, including *y*-elongated stripe vortices, can be expressed as

6 $$P=\left(P_{1}+P_{a}\sin\frac{\pi z}{h}\sin\frac{\pi x}{d}\right)e_{x}+P_{2}e_{y}+P_{3}\cos\frac{\pi z}{h}\cos\frac{\pi x}{d}e_{z}$$

Here, *d* is the domain width, and **e**_*x*_, **e**_*y*_, **e**_*z*_ are the unit vectors along the *x*-, *y*-, *z*-axes, respectively. Importantly, the *z*-component of the polarization vanishes at the surfaces *z* = ± *h*/2, resulting in the absence of surface bound charges. Furthermore, we take $P_{a}=P_{3}d/h$ to satisfy the condition div**P** = 0, which eliminates the volume bound charges, ρ = −div**P**, and results in vanishing of the unfavorable depolarization energy. This constraint refers to the divergence-free description of polarization fields in ferroelectrics, revealing their fundamental topological properties.^12,58^ Expression [Disp-formula eq6] also allows estimating inhomogeneous polarization-induced strains, superimposed on the surface-tension-induced uniform strain. These strains can be approximated as *δu*_*ij*_ ≈ *Q*_*ijkl*_ *P*_*k*_ *P*_*l*_. The average of *δu*_*ij*_ partially compensates the surface-tension-induced tetragonality.

The GLD energy of nonuniform textures is calculated by substituting **P**(**r**) from eq 6 into the energy density eq 5 and integrating over the film volume. Due to the spatial inhomogeneity of the polarization, the gradient energy contribution, $F_{grad}=\frac{1}{2}G_{ijkl}\left(\partial_{i}P_{j}\right)\left(\partial_{k}P_{l}\right)$ , should also be included. Importantly, the resulting energy is a function of the coefficients $\left\{P_{1},P_{2},P_{3}\right\}$ , further considered as variation parameters. Taking the gradient coefficients *G*_*ijkl*_ from ref 59 for BTO and ref 60 for PTO, we minimize the energy with respect to $\left\{P_{1},P_{2},P_{3}\right\}$ , identifying phases that emerge at different values of stress σ_*s*_ and temperature *T*. Taking into account the dependence (eq 3) of σ_*s*_ on film thickness *h*, we construct *h*–*T* phase diagrams, shown in Figure 3a for BTO and Figure 3b for PTO.

**Figure 3:**
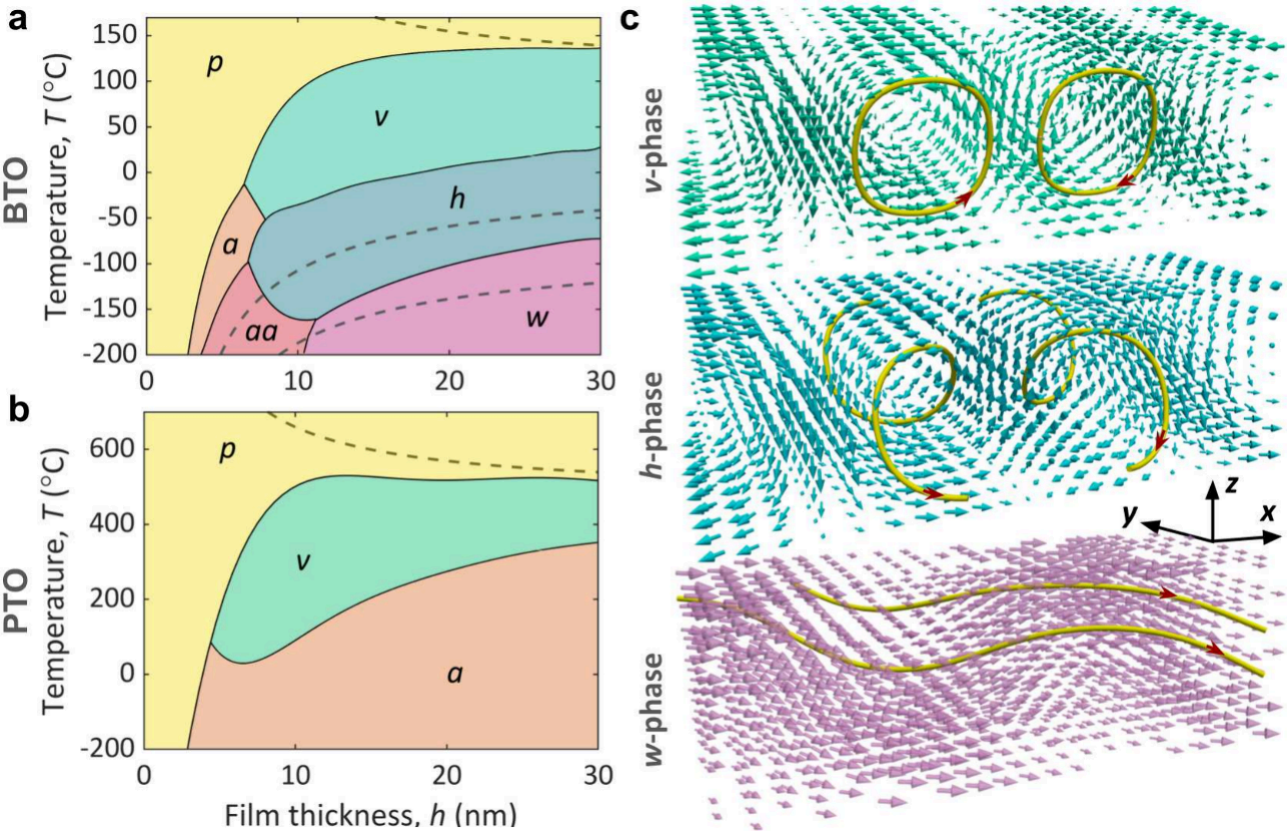
Vortex polarization phases in open-circuit BTO and PTO thin films. (a, b) Thickness–temperature phase diagrams of freestanding BTO (a) and PTO (b) films driven by the surface tension. Dashed lines correspond to the uniform phases shown in Figure 2e and f. (c) Structure of the polarization field in emerging nonuniform *v*-, *h*-, and *w*-phases. Exemplary polarization streamlines are shown in yellow.

Three nonuniform polarization states emerge sequentially as the temperature decreases from the paraelectric *p*-phase in BTO films with thicknesses above 8–10 nm, see Figure 3a.

(i) Phase $\left\{0,0,P_{3}\right\}$ , where only parameter $P_{3}$ in eq 6 is nonzero. Visualization of the polarization identifies this state as vortex *v*-phase, hosting straight elongated vortices with alternating clockwise and counterclockwise polarization rotation, see Figure 3c. This phase corresponds to the vortex phase, observed in PbTiO_3_/SrTiO_3_ (PTO/STO) superlattices^57^ and having the structure of the soft domains.^56^

(ii) Phase $\left\{0,P_{2},P_{3}\right\}$ , where permanent *y*-directed component $P_{2}$ is superimposed on the vortex texture. In this helix *h*-phase, alternating vortices acquire a helicoidal structure, with their cores having the same axially oriented polarization, see Figure 3c. A similar state has also been observed in PTO/STO superlattices.^61^

(iii) Phase $\left\{P_{1},P_{2},P_{3}\right\}$ , where *x*- and *y*-directed components with nearly equal amplitudes $P_{2}\approx P_{1}$ are superimposed on the vortex texture. This *w*-phase has a polarization wave structure, with polarization streamlines propagating approximately along the [110] direction. The presence of all three components $P_{1}$ , $P_{2}$ , and $P_{3}$ makes it analogous to the strain-induced monodomain *r*-phase or stripe-domain phase with the polarization rotated away from the tetragonal axis.^62^ A similar *w*-phase has been observed in PTO/STO superlattices.^51^

In addition, two uniform states, *a*-phase (*P*_1_, 0, 0) and *aa*-phase (*P*_1_, *P*_1_, 0), emerge in films with thicknesses below 8–10 nm. Their energies are competitive to those of the nonuniform phases, and the phase boundary between uniform and nonuniform states shifts to higher thicknesses as γ decreases. Notably, polar phases are suppressed in ultrathin films.

In contrast to the rich phase diagram of BTO, PTO exhibits only two ordered phases upon cooling from the paraelectric state (Figure 3b): vortex-like *v*-phase $\left\{0,0,P_{3}\right\}$ and uniform *a*-phase (*P*_1_, 0, 0). The variety of nonuniform phases in BTO reflects its cascade of ferroelectric transitions under short-circuit conditions, shown in Figure 2a,c,e. The simpler phase diagram of PTO is explained by its stronger uniaxial anisotropy, which already limits the diversity of polar configurations in the uniform regime.

Remarkably, the *v*-phase, described by a single parameter, corresponds to the uniform *c*-phase; *h*-phase (two parameters) mirrors the *ac*-phase; and *w*-phase (three parameters) reflects the *r*-phase. The sequences of the nonuniform phases observed upon cooling in Figure 3a and b follow the order of their uniform counterparts in Figure 2e and f, indicated in Figure 3a and b by dashed lines.

In the study of surface-tension-induced topological states, we focused on straight, stripe-like vortex configurations. However, polarization textures may form more complex patterns, such as bubble-like structures^63,64^ or labyrinthine domains,^65^ viewed as superpositions of multiple wavevectors in (6). Their formation is governed by a subtle interplay of additional energy contributions, including nonuniform electrostrictive and flexoelectric couplings, higher-order gradient terms, residual depolarization fields, and boundary conditions not fully captured within the soft-domain framework. While the overall phase diagram structure is expected to remain largely intact, nonuniform patterns within each phase may vary, giving rise to other intricate polarization textures. Furthermore, in BTO, the extremely small energy difference between polar phases makes ferroelectric ordering highly sensitive to the precise value of γ, potentially leading to the coexistence of metastable clusters associated with different states, resulting in a chaotic, relaxor-like behavior.

Beyond internal polarization states, surface tension can also induce large-scale morphological instabilities in freestanding films, such as bending, folding, and wrinkling.^66–69^ These deformations can be suppressed by laminating freestanding films onto stretchable polymers, enabling homogeneous strain control and strain-induced ferroelectricity, as demonstrated in SrTiO_3_ membranes.^70^ The instabilities arise when the surface-tension-induced compressive stress exceeds the bending rigidity, leading to spontaneous out-of-plane undulations that reduce the elastic energy of the system.

We analyze this Euler-type instability in a freestanding film of thickness *h* and lateral dimensions *L* × *L*, subject to bending along the *x*-axis with vertical displacement $u_{z}=\frac{1}{2}Ax^{2}$ and corresponding shear strain *u*_*zx*_ = *Ax*. The bending energy for small deformations in the isotropic case is given by elasticity theory^34^ as $U_{bend}=\frac{1}{2}DL\int_{0}^{L}{\left(\partial_{x}u_{zx}\right)}^{2}dx=\frac{1}{2}DA^{2}L^{2}$ , where the bending rigidity is *D* = *Eh* ^3^/12­(1 – ν^2^), with *E* the Young’s modulus and ν the Poisson’s ratio. For pseudocubic materials, it becomes $D=h^{3}\left(C_{11}^{2}-C_{12}^{2}\right)/12C_{11}$ .

The surface energy gain due to the excess area under deformation is $U_{\gamma }=-\gamma \Delta S\approx -\frac{\gamma }{3}A^{2}L^{4}$ , where the surface excess at *u*_*zx*_ ≪ 1 is $\Delta S=2L\int_{0}^{L}\left(\sqrt{1+u_{zx}^{2}}-1\right)dx\approx L\int_{0}^{L}u_{zx}^{2}dx=\frac{1}{3}A^{2}L^{4}$ . The instability occurs when the total energy *U*_bend_ + *U*_γ_ becomes negative, providing the critical thickness

7 $$h_{cr}={\left(\frac{8\gamma L^{2}C_{11}}{C_{11}^{2}-C_{12}^{2}}\right)}^{1/3}$$

For a lateral film size *L* = 5 mm, this yields an estimate *h*_cr_ ≈ 13 μm in BTO and *h*_cr_ ≈ 17 μm in PTO. This result indicates that freestanding films with millimeter-scale lateral dimensions and ∼10 μm thickness may spontaneously wrinkle or buckle under surface tension, introducing structural complexity that may interact with polarization patterns. Bending partially relaxes the surface-induced compressive stress, slightly shifting phase boundaries toward smaller thicknesses in *h*–*T* diagrams. Additionally, stress modulation from wrinkling may induce coexistence of different types of polarization domains across the film, further enriching the complexity of its phase behavior.^71^

Morphological instabilities in freestanding ferroelectric films, such as bending and wrinkling, have been observed and attributed to several distinct mechanisms, including flexoelectric coupling and electrostatic boundary effects.^71–74^ Their impact on the formation and stability of polar topological structures has been discussed recently,^71^ providing valuable insights into ferroelectric thin-film electromechanics. Here, we propose a universal mechanism based on the competition between surface tension and bending rigidity, independent of the ferroelectric order and applicable in the paraelectric phase. Thus, bending or wrinkling observed above the Curie temperature would strongly support surface-tension-induced deformation as a mechanism for topological phase formation in freestanding ferroelectric films.

To conclude, freestanding ferroelectric films open a new regime where surface-tension-induced elastic forces become the primary driver of ferroelectric behavior. We have shown that the resulting uniform compressive stress can stabilize both uniform and topologically nontrivial polarization states, offering an alternative route to phase engineering beyond epitaxial strain. Surface tension can also trigger large-scale morphological instabilities, such as bending and wrinkling, which may additionally alter the system’s phase diagram. Furthermore, surface tension is likely to play an important role in epitaxial films by acting on the free surface and contributing to the overall stress balance alongside substrate-induced strain.
